# Supplementary material for: Chronic nicotine increases midbrain dopamine neuron activity and biases individual strategies towards reduced exploration in mice
Source: Nat Commun. 2021 Nov 26;12:6945. doi: 10.1038/s41467-021-27268-7 (PMC8635406; doi:10.1038/s41467-021-27268-7)
Supplement: Supplementary file 1 — Supplementary information. [file 41467_2021_27268_MOESM1_ESM.pdf]

Supplementary Information

Chronic nicotine increases midbrain dopamine neuron activity and biases individual strategies towards reduced exploration. Malou Dongelmans, Romain Durand-de Cuttoli, Claire Nguyen, Maxime Come, Etienne K. Duranté, Damien Lemoine, Raphael Brito, Tarek Ahmed Yahia, Sarah Mondoloni, Steve Didienne, Elise Bousseyrol, Bernadette Hannesse, Lauren M. Reynolds, Nicolas Torquet, Deniz Dalkara, Fabio Marti, Alexandre Mourot, Jérémie Naudé, Philippe Faure

Type	Sequence / Reference	Concentration	Provenance	Use
(Virus) Floxed CatCh	AAV5.Ef1α.DIO.hCatCh.YFP	2.46e^{12} - 6.53e^{13} vg/ml	Institut de la Vision (D. Dalkara)	Fig.5, Fig.Sup.6
(Virus) Floxed Jaws	AAV5.Ef1α.DIO.Jaws.eGFP	1.16e^{13} vg/ml	Institut de la Vision (D. Dalkara)	Fig.5, Fig.Sup.6
(Virus) Floxed YFP	AAV5.Ef1α.DIO.YFP	6.89e^{13} or 9.10e^{13} vg/ml	Institut de la Vision (D. Dalkara)	Fig.5, Fig.Sup.6
(Antibody) Ab1: Mouse anti-TH	T1299	1:500 dilution	Sigma	Fig.5, Fig.Sup.6
(Antibody) Ab2: Cy3 anti-mouse	715-165-150	1:500 dilution	Jackson ImmunoResearch	Fig.5, Fig.Sup.6
(Antibody) Ab1: Chicken anti-YFP	A-6455	1:1000 dilution	Life technologies Molecular Probes	Fig.5, Fig.Sup.6
(Antibody) Ab2: AlexaFluor488 anti-chicken	711-225-152	1:1000 dilution	Jackson ImmunoResearch	Fig.5, Fig.Sup.6

Supplementary Table 1: List of all viruses and antibodies

Supplementary Figure 1: **Stimulating electrode implantation:** Representative examples of unilateral MFB implantations in two different brains. Post-hoc verification of the ICSS track is represented in dotted yellow line.

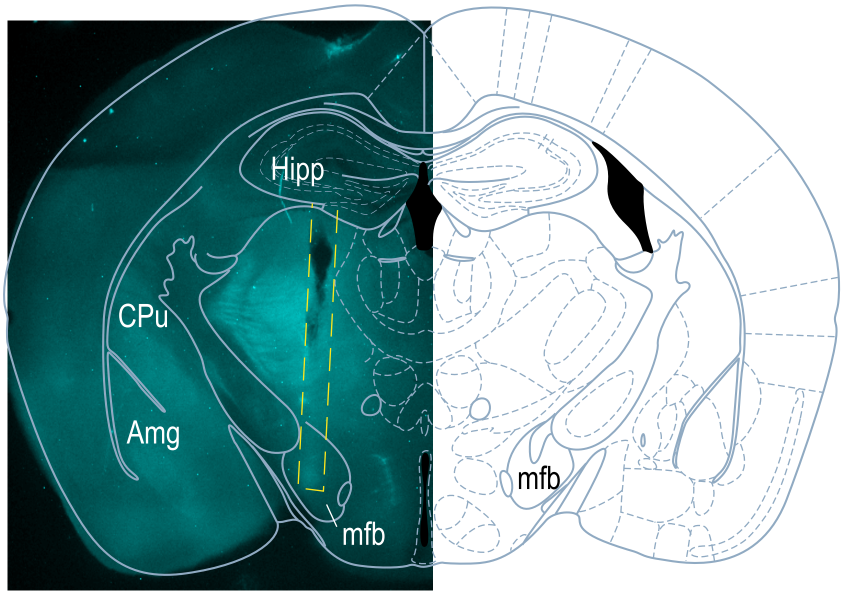

Bregma -1.34 mm
Yellow (ICSS)

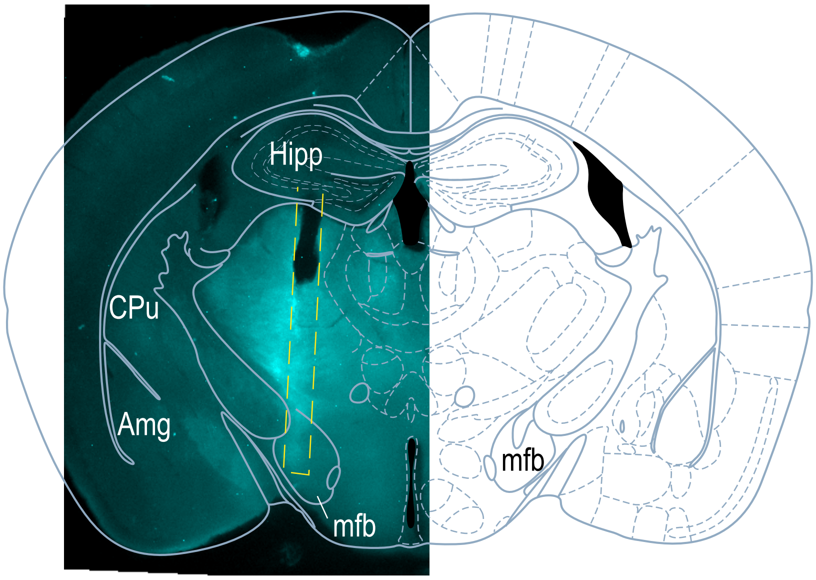

Bregma -1.34 mm
Yellow (ICSS)

Supplementary Figure 1

Supplementary Figure 2: **a-c No behavioral difference between mice implanted with an osmotic minipump filled with saline (Sal) and non-implanted mice. a** Comparison of the percentage of directional change in Sal (black, n = 23) and non-implanted (grey, n = 32) mice (two-sided Wilcoxon signed rank test, p =0.321). **b** Repartition of the visits to the three targets in the probabilistic setting (PS) (two-sided Wilcoxon Test p =0.5, 0.77 and 0.8 for the three comparisons). **c** Probability to choose the alternative with the highest probability of reward for the three possible gambles: G_{100}; G_{25} and G_{50} (two-sided Wilcoxon Test p=0.64,1 and 0.36 for G_{100}, G_{50} and G_{25}). **d Nicotine-treated mice show increased locomotion for the first five minutes in an open field.** Top, trajectory in an open field (duration 30 minutes) of a mouse treated for 24 days with nicotine (10 mg/kg/day). Bottom, cumulative distance travelled in meters measured every 5 minutes during a 30 min-OF exploration. Nic mice (n = 14) showed a greater distance travelled during the first 5 minutes only (two-sided Student's t-test, p = 0.031), compared to saline-exposed mice (Sal, n = 12). The total distance travelled after 30 minutes was not significantly different between the two groups (p =0.397). All data are shown as mean ± sem.

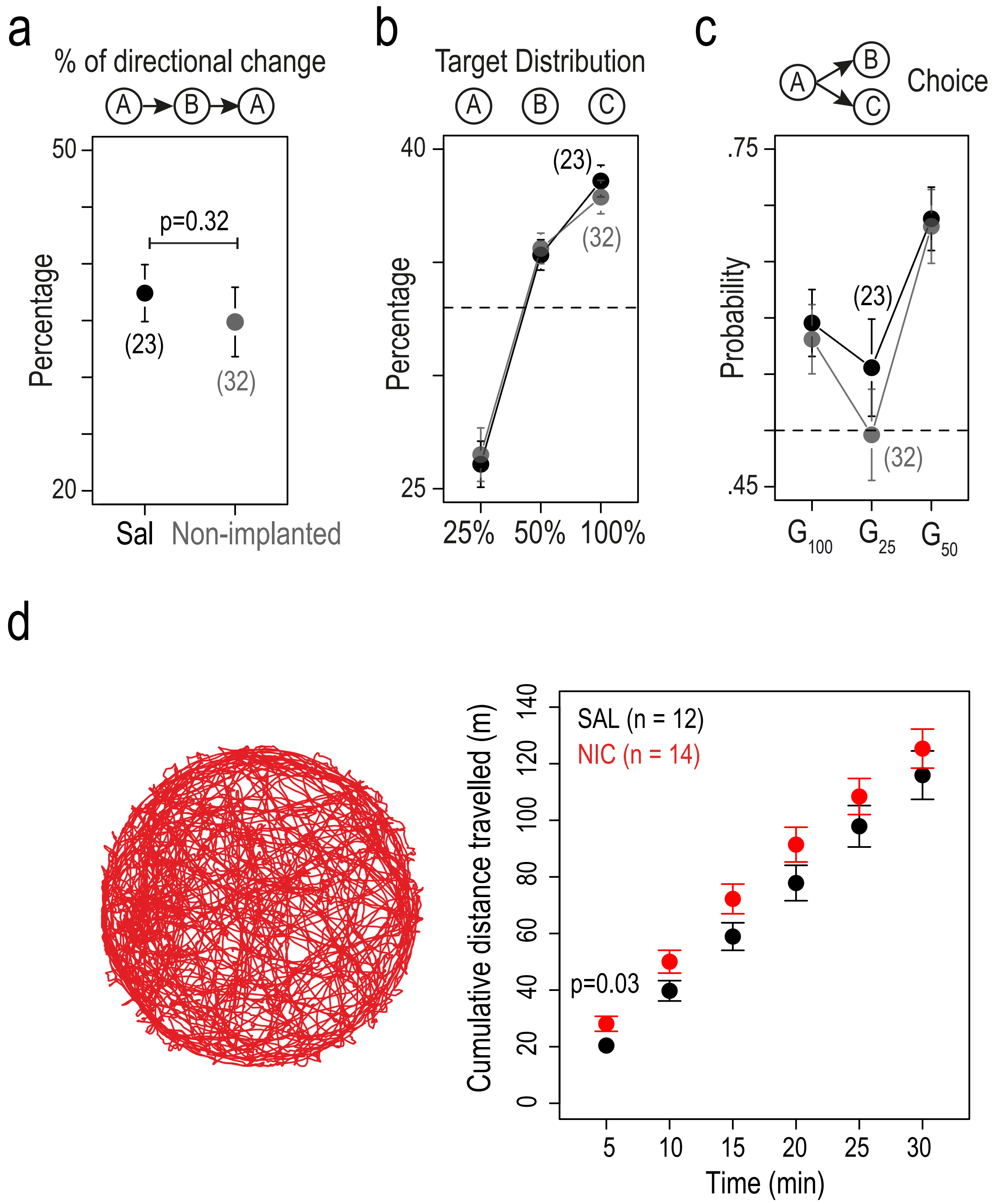

Supplementary figure 2

Supplementary Figure 3: **Archetypal analyses with 2 to 4 apices**: Archetypal analysis of the choice strategies based on a 7-dimensional data space: % of directional change, Gambles G_{100} (choice 50% over 25%), G_{25} (100% over 50%) and G_{50} (100% over 25%), and probabilities of choosing each point (P_{25}, P_{50}, and P_{100}). Analyses were performed on n = 82 mice (pooled control and nicotine mice). **a** Residual sum of squares for a number or archetypes n_A = 1 to 10. Error reduction between n_A = 4 and 10 is marginal. **b** Plot of the archetypal solutions for n_A = 3. Left: Percentile plot of the value of the 7 basic variables used in this analysis for the three archetypes, here labelled A_{3-1} to A_{3-3}. A_{3-1}, A_{3-2} and A_{3-3} correspond to the GO, Des and Asc archetypes of Figure 3d, but are unlabeled here for comparison purposes with n_A = 2 and 4. Right: Visualization of the α coefficients using a ternary plot, in which the three apices represent the three archetypes. Each point shows the projection of each individual (n=82). Points are color-coded according to their proximity to the archetypes. **c** Plot of the archetypal solutions A_{2-1} and A_{2-2} for n_A = 2. From left to right: Visualization of the α coefficients in a binary plot, percentile plot and ternary plot (same as in B, with points color-coded according to their proximity to the two archetypes A_{2-1} and A_{2-2}). **d** Same as C for n_A = 4 (A_{4-1} to A_{4-4}). Note that the A_{3-3} (a.k.a. GO) archetype is present when both n_A = 2 (the A_{2-1} archetype) and n_A = 4 (A_{4-2}).

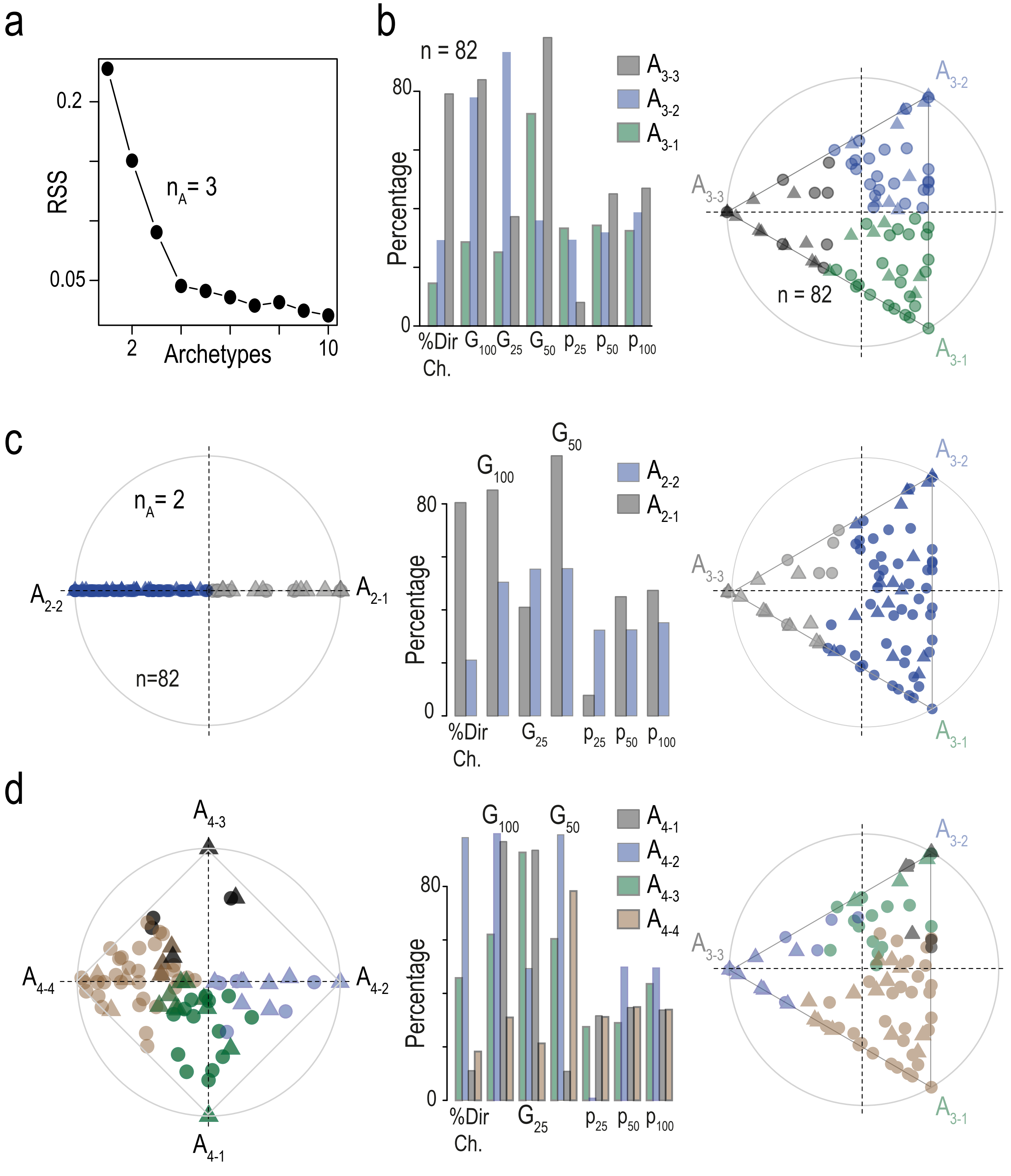

Supplementary Figure 3

Supplementary Figure 4: **Evolution of choice behavior parameters with session. a** Adaptation to the task in the probabilistic setting (PS) with convergence to the mean value (Dashed lines) after few sessions for the success rate, the probability to choose the option with the highest probability of reward in gamble G_{25}, the directional change and the percentage of visits to the 25% target (P_{25}). Mean values (dashed lines) were obtained by means of last two days of probabilistic setting for each individual (n = 82, all animals with pooled 55 control and 27 nicotine-treated mice). **b** Individual values (each point represents one mouse in a given session) for the directional change (above), the success rate (Middle) and the percentage of visits to the 25% target (P_{25}). Black lines indicate linear regression model between session and individual values for session higher than 4 (red background). Estimated slope value (a), probability (p) to reject the null hypothesis of a null slope and the square of the correlation coefficient (R^2). This data indicates no change of value with session number after the first 5 sessions (n = 55 for control mice). Sessions > 10th can have fewer than 55 individual data points. **c** Distribution (n=10000 estimates) of the absolute value of the difference in directional change (return, above) and in the percentage of visits to the 25% target (P_{25}, below) for 2 sessions (higher than the fifth session) in different animals. Vertical dashed line indicated the 1‰ quantile. Vertical red line indicates the mean of the absolute value of the difference estimated between consecutive sessions within the same animals. Inter-individual variations are lower than the intra-individual variations.

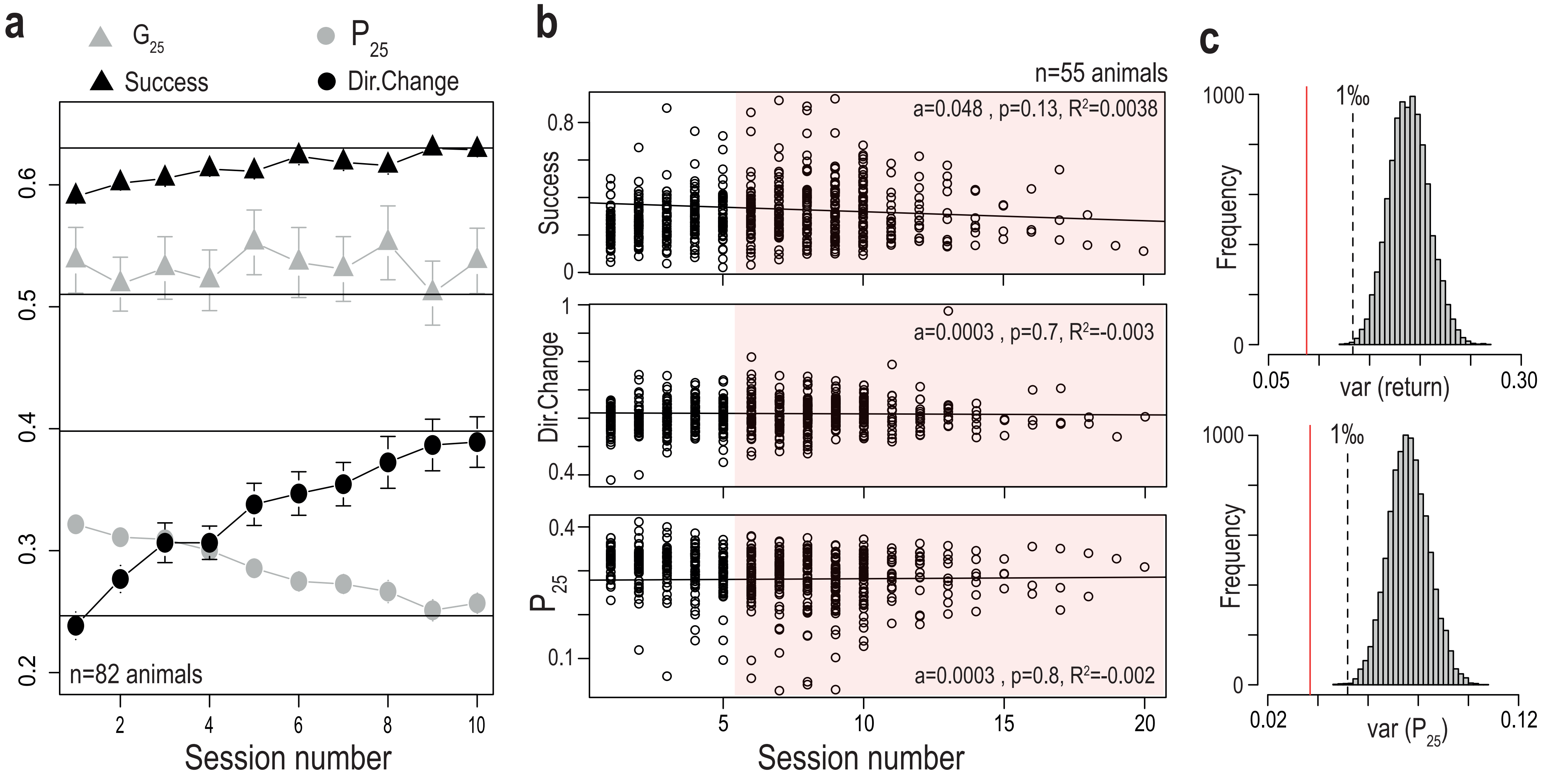

Supplementary Figure 4

Supplementary Figure 5: Principle of the softmax model: a Softmax decision rule with three parameters β (inverse temperature or exploration/exploitation), φ (uncertainty bonus) and κ (cost or effort for a directional change). Three models can be derived from the main model labelled M3 for which ($\beta>0$, $\varphi>0$, $\kappa>0$) M1 and M2 are two special cases of M3. M1: $\beta=0$, $\varphi=0$, $\kappa>0$, M2: $\beta>0$, $\varphi=0$, $\kappa>0$ (see Methods). The table indicates the range of values for the three parameters depending on the model M1, M2 or M3. **b** Model sequence of 2000 choices (n = 55 to match non-exposed mice sample size) simulated with fitted values of β, φ and κ for M1, M2 and M3 (same data as in Figure 4c for M3) Left: Repartition of visits on the three targets (p_{25}, p_{50} and p_{100}), Middle: percentage of directional changes and Right: Probability to choose alternatives with the highest probability of reward for the three possible gambles (G_{100} = p_{50} over p_{25}; G_{25} = p_{100} over p_{50}; G_{50} = p_{100} over p_{25}). Data shown as mean ± sem. **c** Density of twice the difference in log likelihoods of M1 and M3 (blue line) and of M1 and M3 (red line). Vertical dashed line indicates critical d value (d_c) for 95% significance of ratio test (see Methods). Colored horizontal line indicates number of d>dc for each comparison. For b data is shown as mean ± sem. Boxplot shown median, quartiles, and extreme values.

a

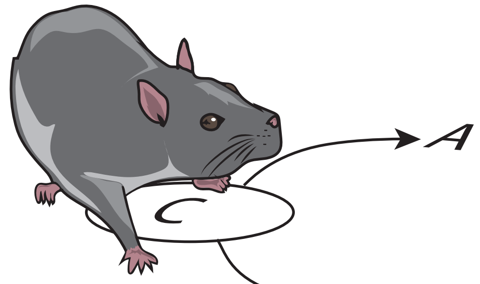

$$\frac{1}{1+e^{-ß(VA-VB)+K}}$$

	β	φ	κ
M3	] 0 , 10]	] 0 , 5]	] 0 , 5]
M2	] 0 , 10]	0	] 0 , 5]
M1	0	0	] 0 , 5]

b

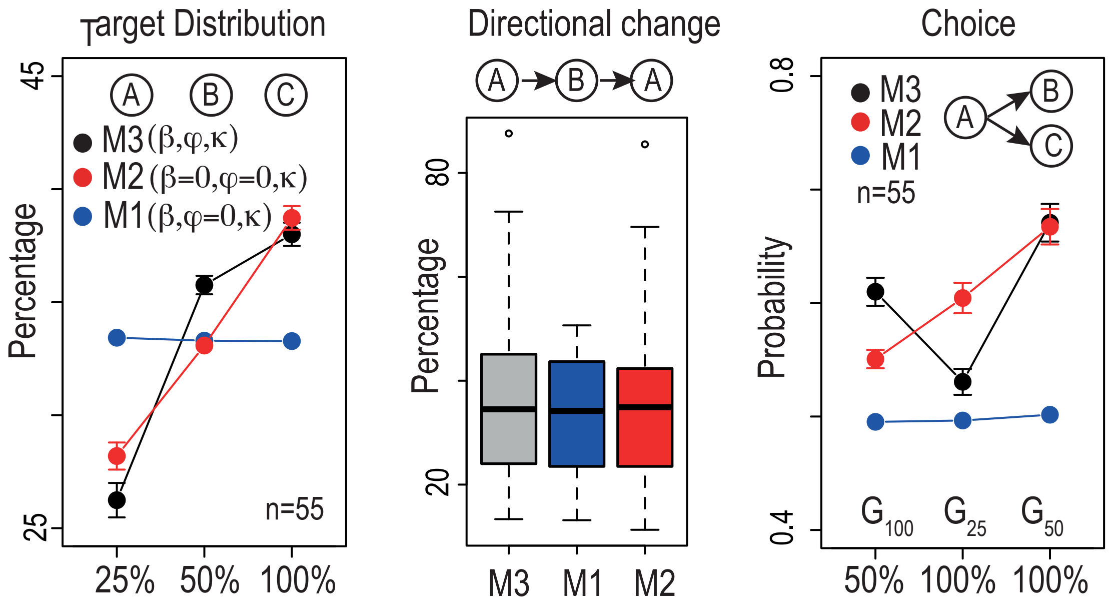

c

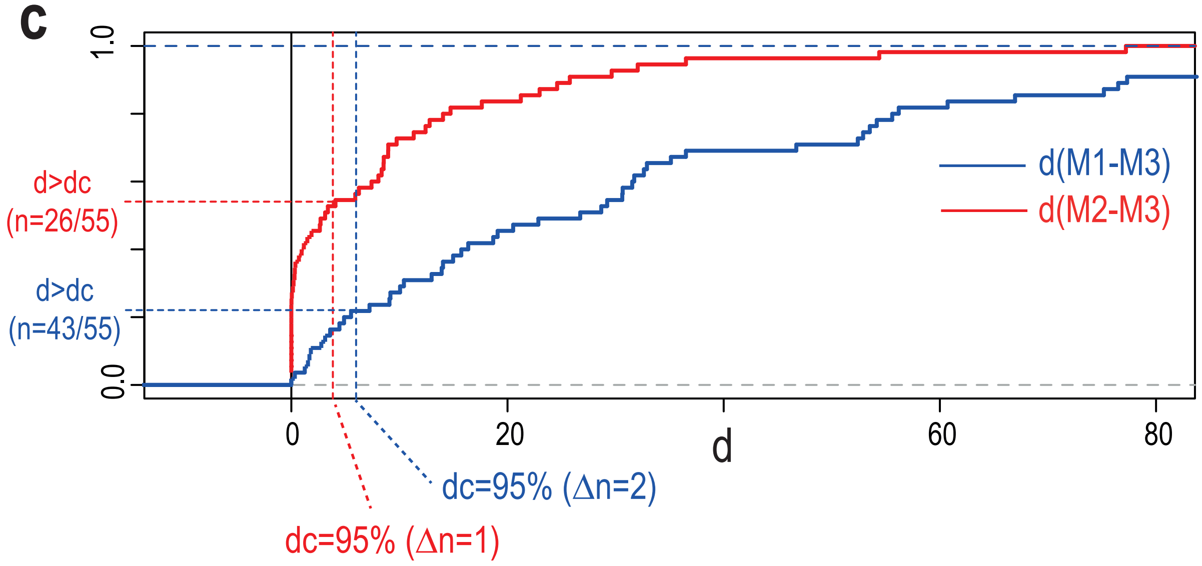

Supplementary Figure 5

Supplementary Figure 6: **Optogenetic manipulation of choice behaviors. a** DAT^{iCRE} mice were implanted unilaterally with bipolar stimulating electrodes for ICSS in the medial forebrain bundle (1) and transduced with either an AAV-DIO-Jaws-eGFP or an AAV-DIO-Catch-YFP in the VTA (2). **b** Representative immunohistochemical verification of CatCh-YFP expression selectively in DA neurons of the VTA (anti-TH in red, and -GFP in green; merge on top). Post-hoc verification of the unilateral fiber implantation is represented by dotted yellow lines. **c** Representative immunohistochemical verification of Jaws-eGFP expression selectively in DA neurons of the VTA (anti-TH in red, and -GFP in green; merge on top). Post-hoc verification of the unilateral fiber implantation is represented by dotted yellow lines. **d** (Left) Percent of choice toward P_{50} in gamble G_{100} and (Right) the time to goal in individual mice (grey points) for CatCh (n=10) and Jaws-transduced (n=11) mice during On and Off sessions. Mean + sem are in black. Red arrows indicate the net effect of nicotine for comparison (unilateral one-sided Student's t-test or Wilcoxon test). **e** Net effect of light stimulation on eight parameters (b values are divided by ten for presentation). Data from OFF sessions were subtracted from data from ON sessions. In red, the effect of nicotine (NIC) is represented for all parameters, as a comparison factor. (Asterisk: chi-squared test to compare the number of parameters that vary in the same direction as nicotine during photostimulation or photoinhibition) (see also Supplementary Table 1). For d, data is shown as mean ± sem.

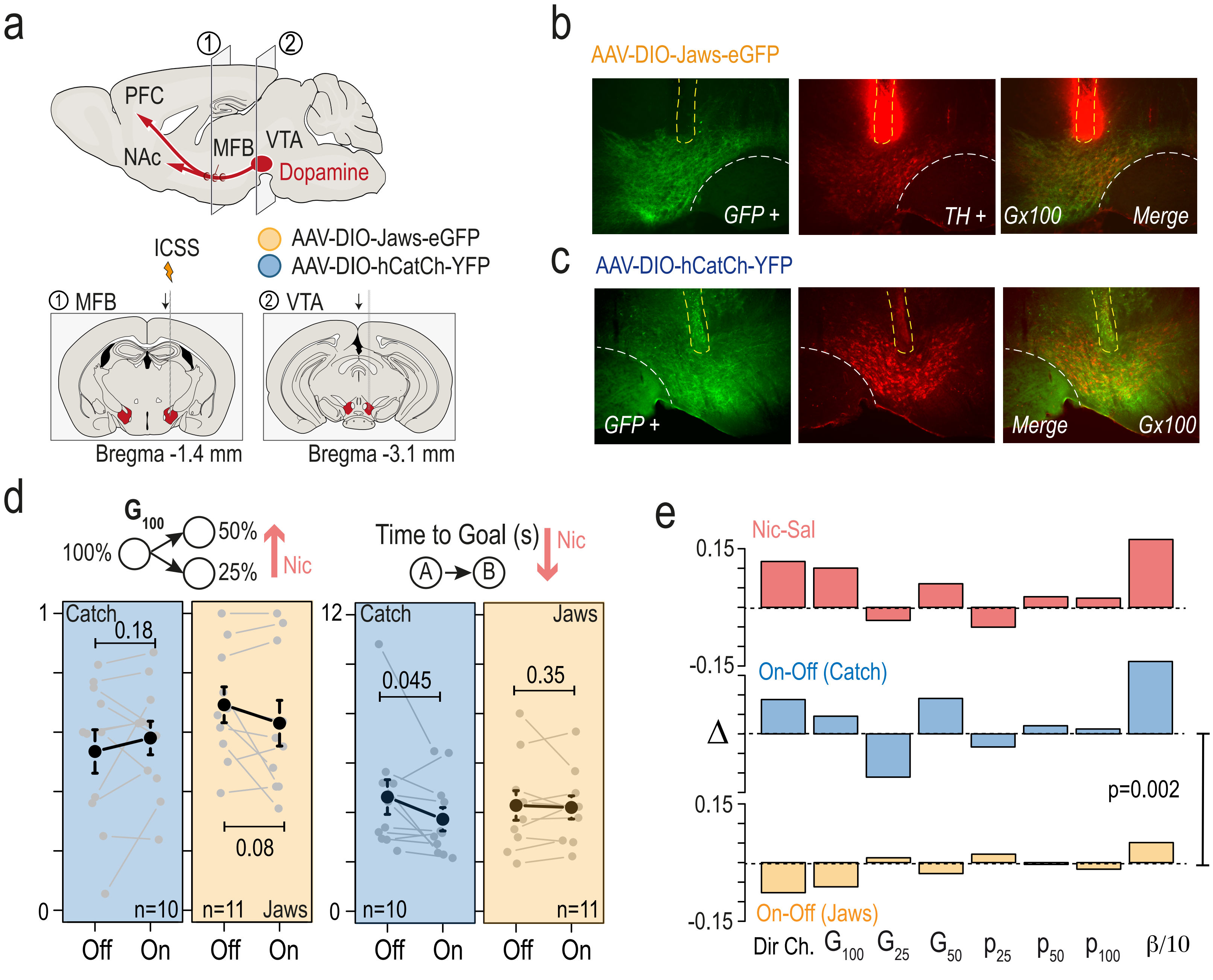

Supplementary figure 6
