# Supplementary material for: Chronic nicotine increases midbrain dopamine neuron activity and biases individual strategies towards reduced exploration in mice
Source: Nat Commun. 2021 Nov 26;12:6945. doi: 10.1038/s41467-021-27268-7 (PMC8635406; doi:10.1038/s41467-021-27268-7)
Supplement: Supplementary file 3 — Reporting summary. [file 41467_2021_27268_MOESM3_ESM.pdf]

nature portfolio

Corresponding author(s): *Double-blind peer review submissions: write DBPR and your manuscript number here instead of author names.*

Last updated by author(s): *YYYY-MM-DD*

Reporting Summary

Nature Portfolio wishes to improve the reproducibility of the work that we publish. This form provides structure for consistency and transparency in reporting. For further information on Nature Portfolio policies, see our Editorial Policies and the Editorial Policy Checklist.

Statistics

For all statistical analyses, confirm that the following items are present in the figure legend, table legend, main text, or Methods section.

n/a	Confirmed	
☐	☒	The exact sample size (*n*) for each experimental group/condition, given as a discrete number and unit of measurement
☐	☒	A statement on whether measurements were taken from distinct samples or whether the same sample was measured repeatedly
☐	☒	The statistical test(s) used AND whether they are one- or two-sided<br>*Only common tests should be described solely by name; describe more complex techniques in the Methods section.*
☐	☒	A description of all covariates tested
☐	☒	A description of any assumptions or corrections, such as tests of normality and adjustment for multiple comparisons
☐	☒	A full description of the statistical parameters including central tendency (e.g. means) or other basic estimates (e.g. regression coefficient) AND variation (e.g. standard deviation) or associated estimates of uncertainty (e.g. confidence intervals)
☐	☒	For null hypothesis testing, the test statistic (e.g. *F*, *t*, *r*) with confidence intervals, effect sizes, degrees of freedom and *P* value noted<br>*Give P values as exact values whenever suitable.*
☐	☒	For Bayesian analysis, information on the choice of priors and Markov chain Monte Carlo settings
☒	☐	For hierarchical and complex designs, identification of the appropriate level for tests and full reporting of outcomes
☒	☐	Estimates of effect sizes (e.g. Cohen's *d*, Pearson's *r*), indicating how they were calculated

Our web collection on statistics for biologists contains articles on many of the points above.

Software and code

Policy information about availability of computer code

Data collection	Animal trajectories are collected with home made labview program
Data analysis	Data are analysed using<br>Python 3.8.5<br>R version 4.1.0

For manuscripts utilizing custom algorithms or software that are central to the research but not yet described in published literature, software must be made available to editors and reviewers. We strongly encourage code deposition in a community repository (e.g. GitHub). See the Nature Portfolio guidelines for submitting code & software for further information.

Data

Policy information about availability of data

All manuscripts must include a data availability statement. This statement should provide the following information, where applicable:

- Accession codes, unique identifiers, or web links for publicly available datasets
- A description of any restrictions on data availability
- For clinical datasets or third party data, please ensure that the statement adheres to our policy

Data Availability: Source data are provided with this paper. All data supporting the findings of this study are available from the corresponding author upon reasonable request.

Code Availability: Animal's trajectories are collected with homemade labview program (version 2014). Results were generated using code written in R (version 4.1.0) and Python (version 3.8.5). All codes used to run the analysis are available from the authors upon request. A sample code for the model and archetype is

publicly available at https://zenodo.org/record/5596424#.YXfBAy8ivgY

Field-specific reporting

Please select the one below that is the best fit for your research. If you are not sure, read the appropriate sections before making your selection.

☒ Life sciences ☐ Behavioural & social sciences ☐ Ecological, evolutionary & environmental sciences

For a reference copy of the document with all sections, see nature.com/documents/nr-reporting-summary-flat.pdf

Life sciences study design

All studies must disclose on these points even when the disclosure is negative.

Sample size	No sample size calculation were performed. Suitable sample sizes were estimated based on previous experiments (Naudé et al Nat Neuro 2016) and are similar to those employed in the field.
Data exclusions	Mice that did not learn the task correctly during the deterministic session were excluded (i.e. they did not self-stimulate above baseline despite increasing stimulation intensity, with a maximum of 200 uA, see Carlezon & Chartoff Nat. Protocols 2007). For the probabilistic sessions, mice self-stimulating at least 35 times in a 5 minutes session were included for the behavioral analysis. We did not exclude animals using statistical outliers.
Replication	All experiments were replicated with sucess. All experiments are at least be replicated twice. The main experiments has been replicated more than 5 times.
Randomization	The probability associated with each target in the probabilistic session was pseudorandomly assigned at the beginning of the probabilistic session and kept constant for each animal. We used C57b6 male mice. Mice that received saline or nicotine where chosen pseudorandomly. For optogenetic experiments, all mice were male DATiCre transgenic mice. Mice that received GFP, Jaws or Catch were chosen pseudorandomly. IFor each experiment the same number of variables were used and analysed.
Blinding	Daily data collection is managed by the software (closed-loop reward delivery upon videotracking), without any investigators action. So blinding is not an issue.

Reporting for specific materials, systems and methods

We require information from authors about some types of materials, experimental systems and methods used in many studies. Here, indicate whether each material, system or method listed is relevant to your study. If you are not sure if a list item applies to your research, read the appropriate section before selecting a response.

Materials & experimental systems

n/a	Involved in the study
☐	☒ Antibodies
☒	☐ Eukaryotic cell lines
☒	☐ Palaeontology and archaeology
☐	☒ Animals and other organisms
☒	☐ Human research participants
☒	☐ Clinical data
☒	☐ Dual use research of concern

Methods

n/a	Involved in the study
☒	☐ ChIP-seq
☒	☐ Flow cytometry
☒	☐ MRI-based neuroimaging

Antibodies

Antibodies used	Mouse anti-TH (Sigma) ; Cy3 anti-mouse (Jackson ImmunoResearch); Chicken anti-YFP (Life technologies Molecular Probes) and AlexaFluor488 anti-chicken (Jackson ImmunoResearch)
Validation	(Antibody) Ab1: Mouse anti-TH<br>https://www.sigmaaldrich.com/FR/fr/product/sigma/t1299<br><br>(Antibody) Ab2: Cy3 anti-mouse<br>No antibody was detected against non-immunoglobulin serum proteins. See details here:<br>https://www.jacksonimmuno.com/catalog/products/715-165-150<br><br>(Antibody) Ab1: Chicken anti-YFP<br>This Antibody was verified by Relative expression to ensure that the antibody binds to the antigen stated. View Details here: https://www.thermofisher.com/antibody/product/GFP-Antibody-Polyclonal/A-6455

(Antibody) Ab2: AlexaFluor488 anti-chicken
No antibody was detected against non-immunoglobulin serum proteins. See details here: https://www.jacksonimmuno.com/catalog/products/711-225-152

Animals and other organisms

Policy information about studies involving animals; ARRIVE guidelines recommended for reporting animal research

Laboratory animals	Male C57BL/6J mouse, and C57Bl/6Rj DATiCRE. 8-16 weeks old.
Wild animals	no wild animals were used in the study
Field-collected samples	no field collected sample were used in the study
Ethics oversight	Experiments were conducted at Sorbonne University, Paris, France, in accordance with the local regulations for animal experiments as well as the recommendations for animal experiments issued by the European Council (directives 219/1990 and 220/1990). The protocol was approved by the Committee on the Ethics of Animal Experiments and the Ministry of Higher Education and Research (permit 01438.02)

Note that full information on the approval of the study protocol must also be provided in the manuscript.
